# Gravity inversion of a fault by Particle swarm optimization (PSO)

**DOI:** 10.1186/2193-1801-2-315

**Published:** 2013-07-15

**Authors:** Reza Toushmalani

**Affiliations:** Department of Computer, Faculty of engineering, Kangavar Branch, Islamic Azad University, Kangavar, Iran

**Keywords:** Gravity inversion, Fault, Particle swarm optimization (PSO)

## Abstract

Particle swarm optimization is a heuristic global optimization method and also an optimization algorithm, which is based on swarm intelligence. It comes from the research on the bird and fish flock movement behavior. In this paper we introduce and use this method in gravity inverse problem. We discuss the solution for the inverse problem of determining the shape of a fault whose gravity anomaly is known. Application of the proposed algorithm to this problem has proven its capability to deal with difficult optimization problems. The technique proved to work efficiently when tested to a number of models.

**Electronic supplementary material:**

The online version of this article (doi:10.1186/2193-1801-2-315) contains supplementary material, which is available to authorized users.

## Introduction

In first section, gravity anomaly produced by fault with known parameters has been calculated and plotted. In Section 2, the Particle swarm optimization (PSO) is proposed and its different parts are studied. In Section 3, the proposed algorithm is tested for the inverse problem of determining the shape of a fault whose gravity anomaly is known. Finally the conclusion is presented in Section 4.

### Appplication to the gravity field of a fault

**Figure 1:**
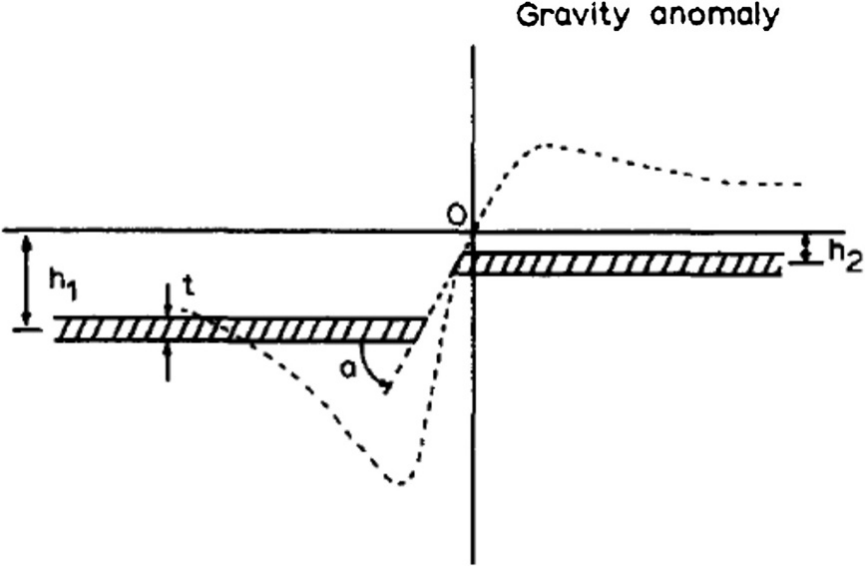
**Fault model illustrating various parameters used in work, and shape of expected gravity anomaly.**

A fault structure can be approximated by two Semi-infinite horizontal sheets, one displaced vertically from the other. The general situation of a fault is presented in Figure 1, together with the shape of the Expected anomaly which is described by the formula (1) (Telford et al. (1976;Thanassoulas et al. 1987):

1 $$g=2kбt\left[\pi +\tan-1\left\{(x/h1+\cot\;\left(a\right)\right\}-\tan-1\left\{(x/h2+\cot\;\left(a\right)\right\}\right]$$

K = 6.672e-3

б = density contrast

t = thickness of sheet

h1,2 = depth of each side to the middle of the sheet

a = fault angle.

### Particle swarm optimization (PSO)

#### Introduction

Optimization is the process of making something better. In other words, optimization is the process of adjusting the inputs to or characteristics of a device, mathematical process, or experiment to find the minimum or maximum output or result. The input consists of variables.

The process or function is known as the cost function, objective function, or fitness function; and the output is the cost or fitness (Haupt & Haupt 2004). There are different methods for solving an optimization problem. Some of these methods are inspired from natural processes. These methods usually start with an initial set of variables and then evolve to obtain the global minimum or maximum of the objective function. Genetic Algorithm (GA) has been the most popular technique in evolutionary computation research.

Genetic Algorithm uses operators inspired by natural genetic variation and natural selection (Melanie 1999; Sivanandam & Deepa 2008). Another example is Particle Swarm Optimization (PSO) which was developed by Eberhart and Kennedy in (Eberhart & Kennedy 1995). This stochastic optimization algorithm is inspired by social behavior of bird flocking or fish schooling (Sivanandam & Deepa 2008) (Engelbrecht 2005)). Ant Colony Optimization (ACO) is another evolutionary optimization algorithm which is inspired by the pheromone trail laying behavior of real ant colonies Sivanandam & Deepa (Dorigo & Blum 2005; Dorigo & Gambardella 1997; Sivanandam & Deepa 2008). On the other hand Simulated Annealing simulates the annealing process in which a substance is heated above its melting temperature and then gradually cools to produce the crystalline lattice, which minimizes its energy probability distribution Haupt & Haupt (De Vicente et al. 2003; Haupt & Haupt 2004; Vecchi Kirkpatrick & Gelatt 1983). Besides these well known methods, the investigations on nature inspired optimization algorithms are still being done and new methods are being developed to continually solve some Sort of nonlinear problems. The novel algorithm integrates advantages of the immune evolutionary algorithm and chaos optimization algorithm. Ahrari et al. (Ahrari et al. 2009) Introduces a new optimization technique called Grenade Explosion Method (GEM) and its underlying ideas, including the concept of Optimal Search Direction (OSD), are elaborated. In a new particle swarm optimization method based on the clonally selection algorithm is proposed to avoid premature convergence and guarantee the diversity of the population. Rajabioun (Rajabioun 2011).

The main advantages of evolutionary algorithms are Sivanandam & Deepa (2008):

1. Being robust to dynamic changes: Traditional methods of optimization are not robust to dynamic changes in the environment and they require a complete restart for providing a solution. In contrary, evolutionary computation can be used to adapt solutions to changing circumstances.
2. Broad applicability: Evolutionary algorithms can be applied to any problems that can be formulated as function optimization problems.
3. Hybridization with other methods: Evolutionary algorithms can be combined with more traditional optimization techniques.
4. Solves problems that have no solutions: The advantage of evolutionary algorithms includes the ability to address problems for which there is no human expertise. Even though human expertise should be used when it is needed and available; it often proves less adequate for automated problem-solving routines. Rajabioun (2011).

##### Particle swarm optimization (PSO)

Particle swarm optimization (PSO) is a population based stochastic optimization technique developed by Dr. Eberhart and Dr. Kennedy in 1995 (Eberhart & Kennedy 1995; Eberhart & Shi 2001; Eberhart & Shi 1998a; Eberhart & Shi 1998b; Kennedy & Eberhart 1995), inspired by social behavior of bird flocking or fish schooling.

PSO shares many similarities with evolutionary computation techniques such as Genetic Algorithms (GA). The system is initialized with a population of random solutions and searches for optima by updating generations. However, unlike GA, PSO has no evolution operators such as crossover and mutation. In PSO, the potential solutions, called particles, fly through the problem space by following the current optimum particles. The detailed information will be given in following sections (http://www.swarmintelligence.org/tutorials.php).

Compared to GA, the advantages of PSO are that PSO is easy to implement and there are few parameters to adjust. PSO has been successfully applied in many areas: function optimization, artificial neural network training, fuzzy system control, and other areas where GA can be applied (http://www.swarmintelligence.org/tutorials.php).

#### Background: Artificial life

The term “Artificial Life” (ALife) is used to describe research into human-made systems that possess some of the essential properties of life. ALife includes two-folded research topic (http://www.alife.org):

1. A Life studies how computational techniques can help when studying biological phenomena
2. A Life studies how biological techniques can help out with computational problems

The focus of this report is on the second topic. Actually, there are already lots of computational techniques inspired by biological systems. For example, artificial neural network is a simplified model of human brain; genetic algorithm is inspired by the human evolution (http://www.alife.org).

Here we discuss another type of biological system - social system, more specifically, the collective behaviors of simple individuals interacting with their environment and each other. Someone called it as swarm intelligence. All of the simulations utilized local processes, such as those modeled by cellular automata, and might underlie the unpredictable group dynamics of social behavior (http://www.alife.org).

Some popular examples are floys and boids. Both of the simulations were created to interpret the movement of organisms in a bird flock or fish school. These simulations are normally used in computer animation or computer aided design (http://www.alife.org; http://www.red3d.com/cwr/boids/).

There are two popular swarm inspired methods in computational intelligence areas: Ant colony optimization (ACO) and particle swarm optimization (PSO). ACO was inspired by the behaviors of ants and has many successful applications in discrete optimization problems (http://iridia.ulb.ac.be/~mdorigo/ACO/ACO.html).

The particle swarm concept originated as a simulation of simplified social system. The original intent was to graphically simulate the choreography of bird of a bird block or fish school. However, it was found that particle swarm model can be used as an optimizer (Yuhui S, James K, Russell C Eberhart SWARM INTELLIGENCE., http://www.engr.iupui.edu/~eberhart/http://www.engr.iupui.edu/~shi/Coference/psopap4.html).

#### The algorithm (http://www.swarmintelligence.org/tutorials.php)

As stated before, PSO simulates the behaviors of bird flocking. Suppose the following scenario: a group of birds are randomly searching food in an area. There is only one piece of food in the area being searched. All the birds do not know where the food is. But they know how far the food is in each iteration. So what’s the best strategy to find the food? The effective one is to follow the bird which is nearest to the food.

PSO learned from the scenario and used it to solve the optimization problems. In PSO, each single solution is a “bird” in the search space. We call it “particle”. All of particles have fitness values which are evaluated by the fitness function to be optimized, and have velocities which direct the flying of the particles. The particles fly through the problem space by following the current optimum particles.

PSO is initialized with a group of random particles (solutions) and then searches for optima by updating generations. In every iteration, each particle is updated by following two “best” values. The first one is the best solution (fitness) it has achieved so far. (The fitness value is also stored.) This value is called pbest. Another “best” value that is tracked by the particle swarm optimizer is the best value, obtained so far by any particle in the population. This best value is a global best and called gbest. When a particle takes part of the population as its topological neighbors, the best value is a local best and is called lbest.

After finding the two best values, the particle updates its velocity and positions with following equation (a) and (b).

a $$v\left[\right]=v\left[\right]+c1*\mathrm{rand}\left(\right)*\left(\mathrm{pbest}\left[\right]‒\mathrm{present}\left[\right]\right)+c2*\mathrm{rand}\left(\right)*\left(\mathrm{gbest}\left[\right]‒\mathrm{present}\left[\right]\right)$$

b $$\mathrm{present}\left[\right]=\mathrm{persent}\left[\right]+v\left[\right]$$

v[] is the particle velocity, persent[] is the current particle (solution). pbest[] and gbest[] are defined as stated before. rand () is a random number between (0,1). c1, c2 are learning factors. usually c1 = c2 = 2.

The pseudo code of the procedure is as follows

For each particle

Initialize particle

END

Do

For each particle

Calculate fitness value

If the fitness value is better than the best fitness value (pBest) in history

set current value as the new pBest

End

Choose the particle with the best fitness value of all the particles as the gBest

For each particle

Calculate particle velocity according equation (a)

Update particle position according equation (b)

End

While maximum iterations or minimum error criteria is not attained

Particles’ velocities on each dimension are clamped to a maximum velocity Vmax. If the sum of accelerations would cause the velocity on that dimension to exceed Vmax, which is a parameter specified by the user. Then the velocity on that dimension is limited to Vmax.

#### PSO parameter control (http://www.swarmintelligence.org/tutorials.php)

From the above case, we can learn that there are two key steps when applying PSO to optimization problems: the representation of the solution and the fitness function. One of the advantages of PSO is that PSO take real numbers as particles. It is not like GA, which needs to change to binary encoding, or special genetic operators have to be used. For example, we try to find the solution for f(x) = ×1^2 + ×2^2 + ×3^2, the particle can be set as (×1, ×2, ×3), and fitness function is f(x). Then we can use the standard procedure to find the optimum. The searching is a repeat process, and the stop criteria are that the maximum iteration number is reached or the minimum error condition is satisfied.

There are not many parameter need to be tuned in PSO. Here is a list of the parameters and their typical values.

The number of particles: the typical range is 20–40. Actually for most of the problems 10 particles is large enough to get good results. For some difficult or special problems, one can try 100 or 200 particles as well.

Dimension of particles: It is determined by the problem to be optimized,

Range of particles: It is also determined by the problem to be optimized, you can specify different ranges for different dimension of particles.

Vmax: it determines the maximum change one particle can take during one iteration. Usually we set the range of the particle as the Vmax for example, the particle (×1, ×2, ×3).

X1 belongs [−10, 10], then Vmax = 20

Learning factors: c1 and c2 usually equal to 2. However, other settings were also used in different papers. But usually c1 equals to c2 and ranges from [0, 4].

The stop condition: the maximum number of iterations the PSO execute and the minimum error requirement. for example, for ANN training in previous section, we can set the minimum error requirement is one mis-classified pattern. the maximum number of iterations is set to 2000. this stop condition depends on the problem to be optimized.

Global version vs. local version: we introduced two versions of PSO. global and local version. global version is faster but might converge to local optimum for some problems. local version is a little bit slower but not easy to be trapped into local optimim. One can use global version to get quick result and use local version to refine the search. (webpage of International Society for Artificial Life; webpage of Ariel Dolan; Background and Update; Web site responsible: Romain Hendrickx and Leonardo Bezerra; Kennedy J, Eberhart RC (1995); PSO Tutorial; Shi & Eberhart 1998).

Another factor is inertia weight, which is introduced by Shi and Eberhart (1998). If you are interested in it, please refer to their paper in 1998. (Title: A modified particle swarm optimizer).

### Application of particle swarm optimization (PSO) in inverse problem solving

**Figure 2:**
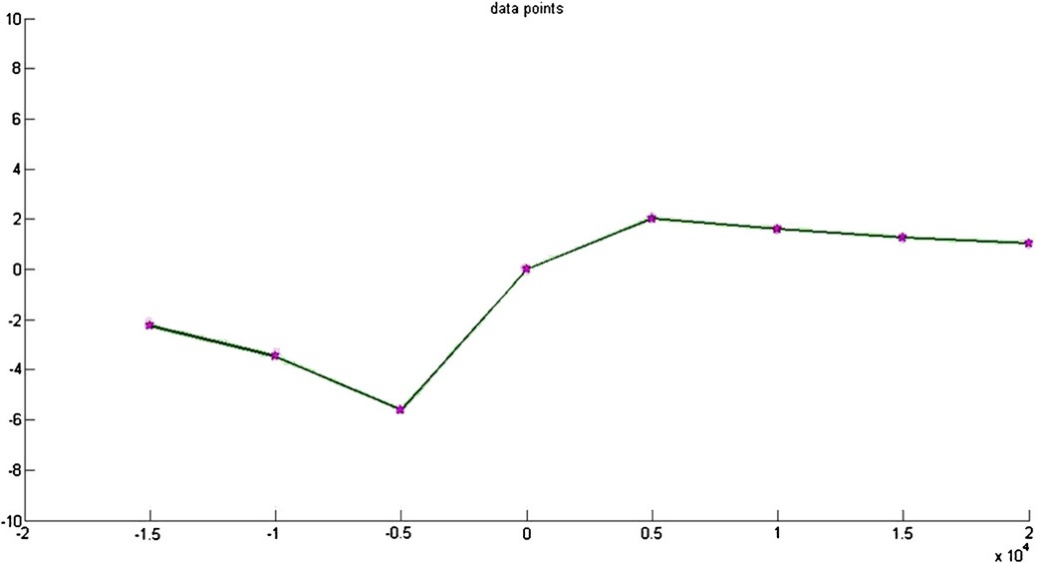
**Theoretical gravity anomaly produced by fault with known parameters and corresponding anomaly (solid triangles) of solution given by gravity inversion program.**

Using Equation (1), the theoretical anomaly which corresponds to a fault with t = 500 m, h1 = 6000 m (left), h 2 = 2000 m, a = 30°, and б = 1, is presented as a continuous line in Figure 2. To test the program, the theoretical anomaly of Figure 2 is digitized every 5000 m (Table 1), and a “bad” initial model with parameters h1 = 3000 m, h2 = 1600 m, t = 700 m, and a = 30° is entered. (Thanassoulas et al. 1987) and Table 2 shows Parameters of obtained solution.

**Table 1:** **Gravity anomaly for inversion**

x-coordinate (m)	Gravity anomaly (mgal)
−15000	−2.24
−10000	−3.47
−5000	−5.60
0	0
5000	2.02
10000	1.61
15000	1.27
20000	1.04

**Table 2:** **Parameters of obtained solution**

Observed gravity	Calculated gravity
−2.24	−2.23
−3.47	−3.47
−5.60	−5.60
0	0
2.02	2.00
1.61	1.63
1.27	1.29
1.04	1.05

h2 = 2001.6431, h1 = 6000 m a = 1.05*π- π =189-180 = 90 , t = 501.44849.

Cost = 3.6519e-005.

During the iterations the density contrast is kept as a fixed parameter, assuming that its value has been estimated previously. The parameters which are optimized are:

- the thickness of the sheet,
- the left distance to the middle of the sheet,
- the right distance to the middle of the sheet, and
- the angle of the fault.

**Figure 3:**
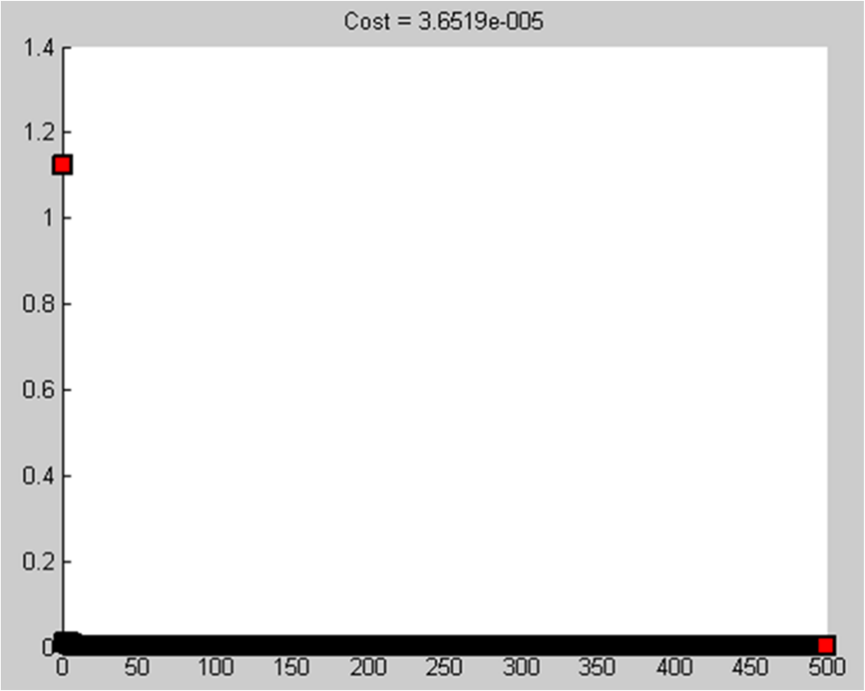
**Cost function value-PSO iteration.**

**Figure 4:**
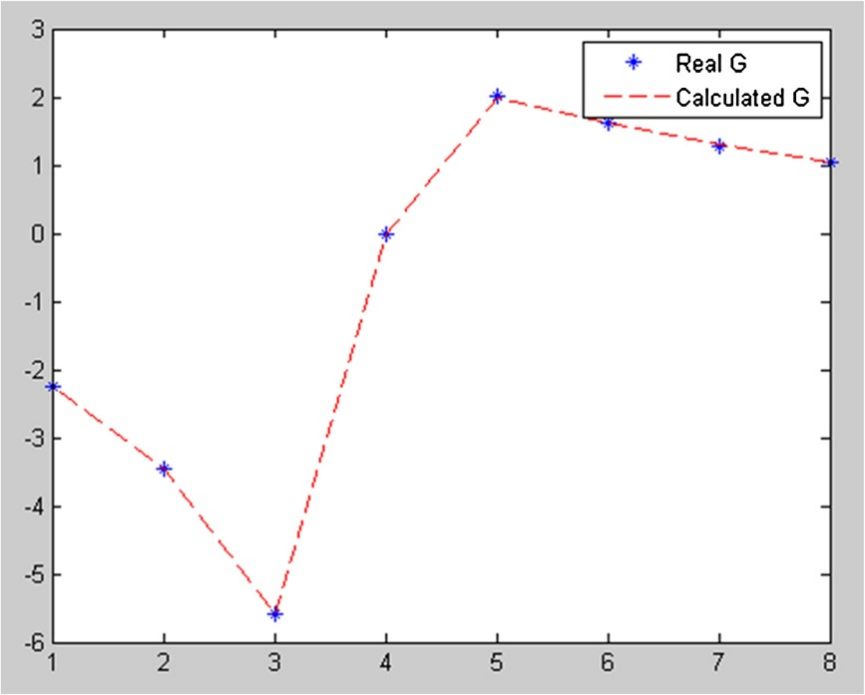
**Observed g-calculated g graph.**

**Figure 5:**
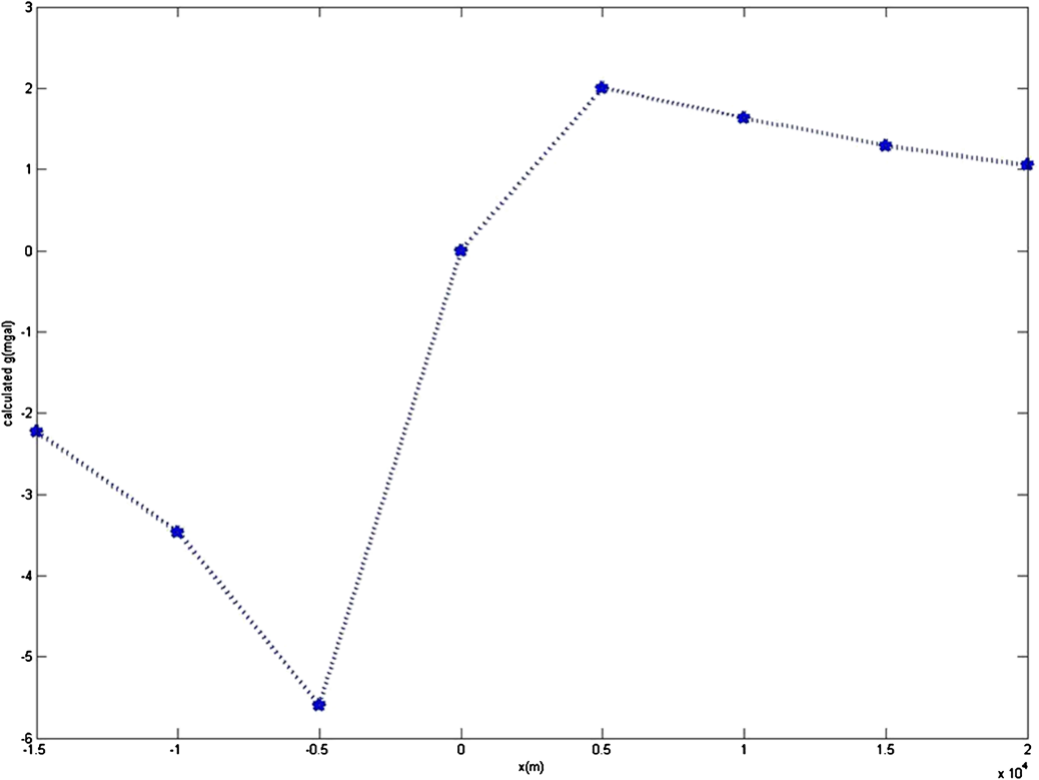
**Calculated g graph.**

PSO set parameters:

CostFunc = g = 2kбt[π + tan-1{(x/h1 + cot (a)}- tan-1{(x/h2 + cot (a)}]

npar = 4;

varLow = 0;

varHigh = 1;

c1 = 2;

c2 = 4-c1;

numOfParticles = 100;

maxNumOfIterations = 500;

Figure 2 shows Theoretical gravity anomaly produced by fault with known parameters and corresponding anomaly (solid triangles) of solution given by gravity inversion program. Figure 3 shows Cost function value-PSO iteration. Figure 4 Observed g-calculated g graph and Figure 5 shows Calculated g graph.

## Conclusion

The parameters which are optimized with this method are: (a) the thickness of the sheet,(b) the left distance to the middle of the sheet,(c) the right distance to the middle of the sheet, and(d) the angle of the fault. Inverse solution reveals that fault model parameters are: depth to top of the fault: 2001.6431 m; depth to bottom of the fault: 6000 m; fault angle: 1.05*π- π =189-180 = 90, thickness of fault : 501.44849 m; which agree quite well with the known results. A good agreement has been found between the predicted model anomaly and the observed gravity anomaly.

## Authors’ original submitted files for images

Below are the links to the authors’ original submitted files for images. Authors’ original file for figure 1 Authors’ original file for figure 2 Authors’ original file for figure 3 Authors’ original file for figure 4 Authors’ original file for figure 5
