# Supplementary figures and images for: Gravity inversion of a fault by Particle swarm optimization (PSO)
Source: Springerplus. 2013 Jul 15;2(1):315. doi: 10.1186/2193-1801-2-315 (PMC3724977; doi:10.1186/2193-1801-2-315)

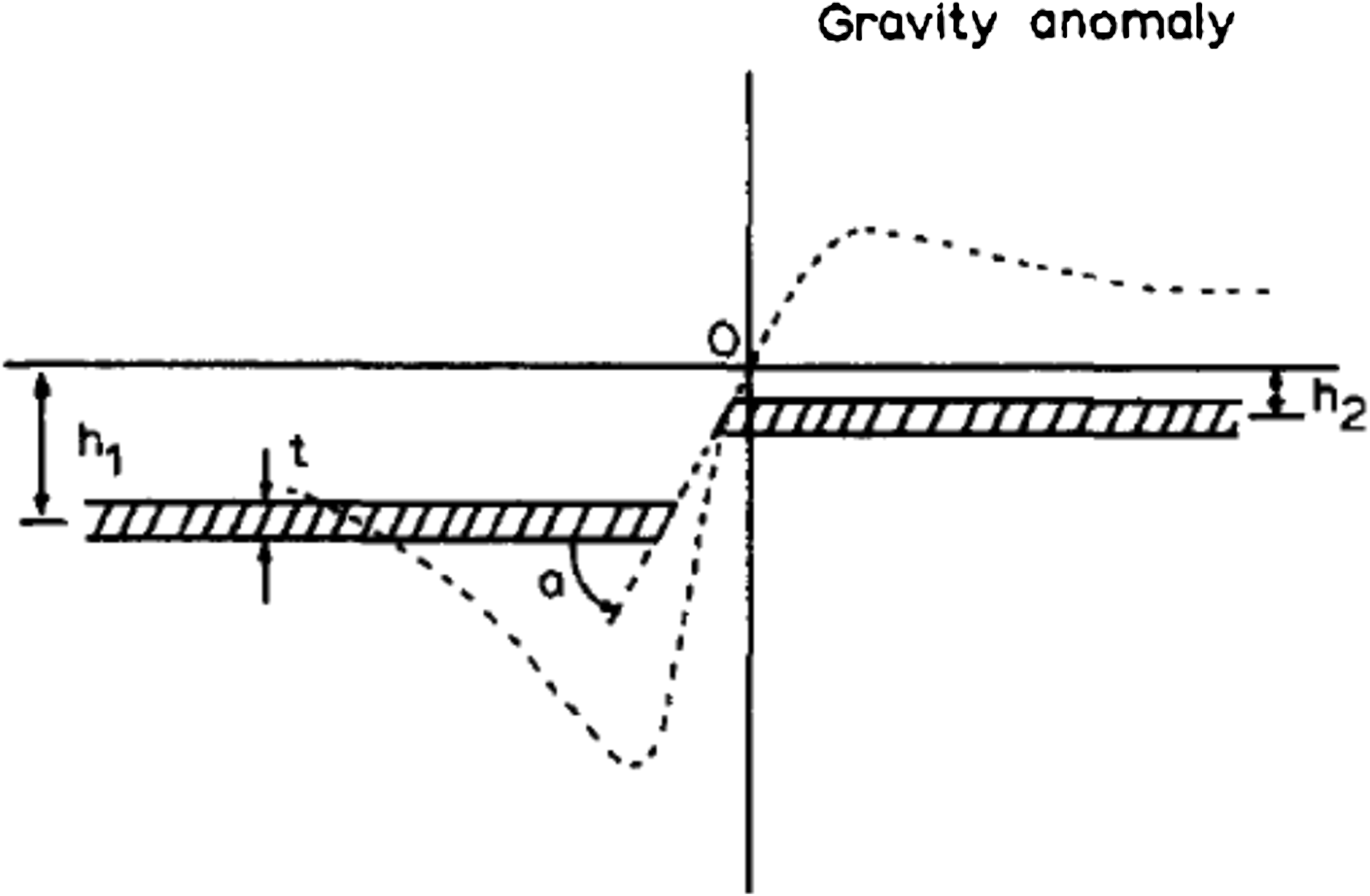

Supplement: Supplementary file 1 — Authors’ original file for figure 1 [file 40064_2013_395_MOESM1_ESM.tif]

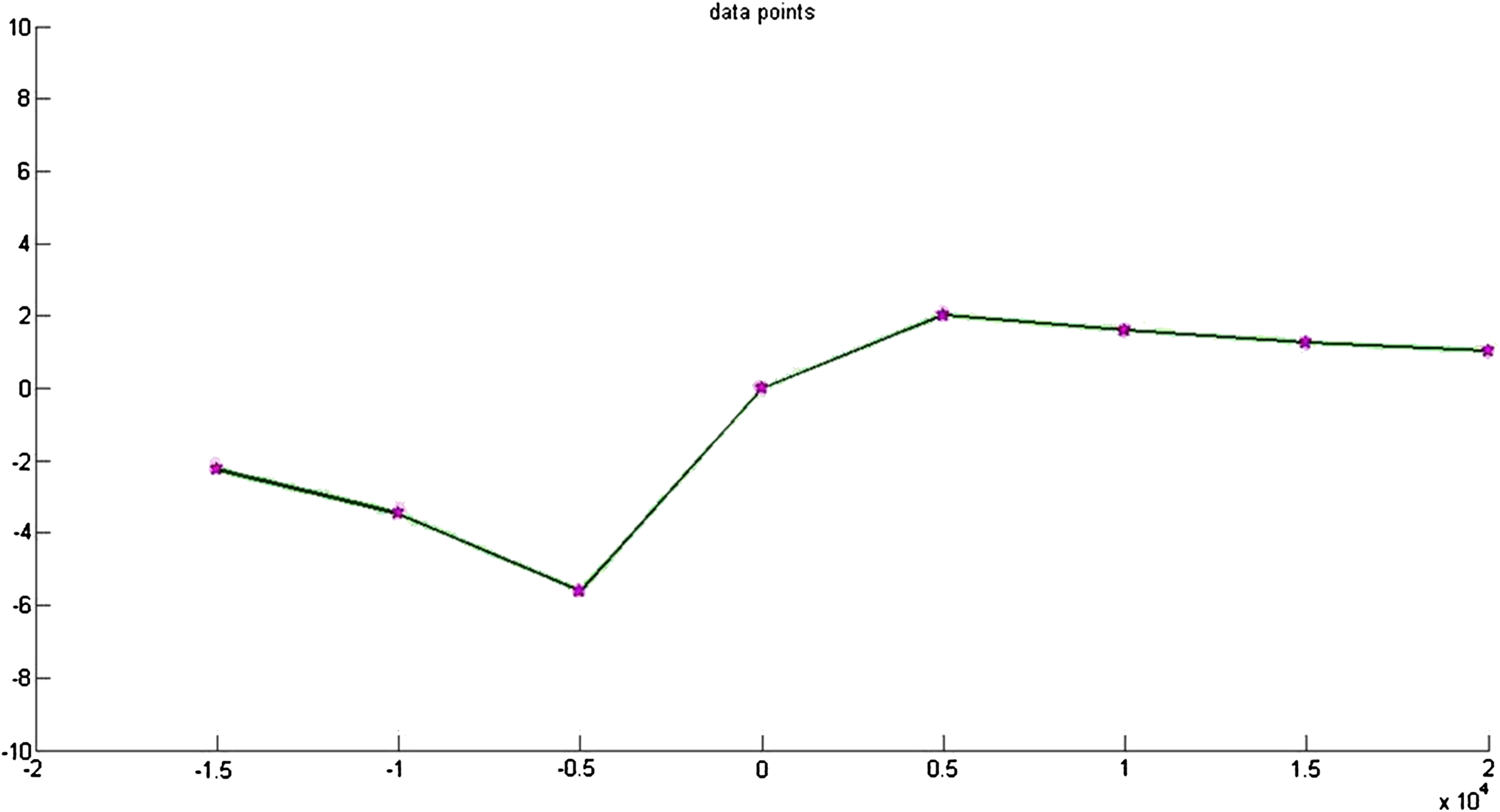

Supplement: Supplementary file 2 — Authors’ original file for figure 2 [file 40064_2013_395_MOESM2_ESM.tif]

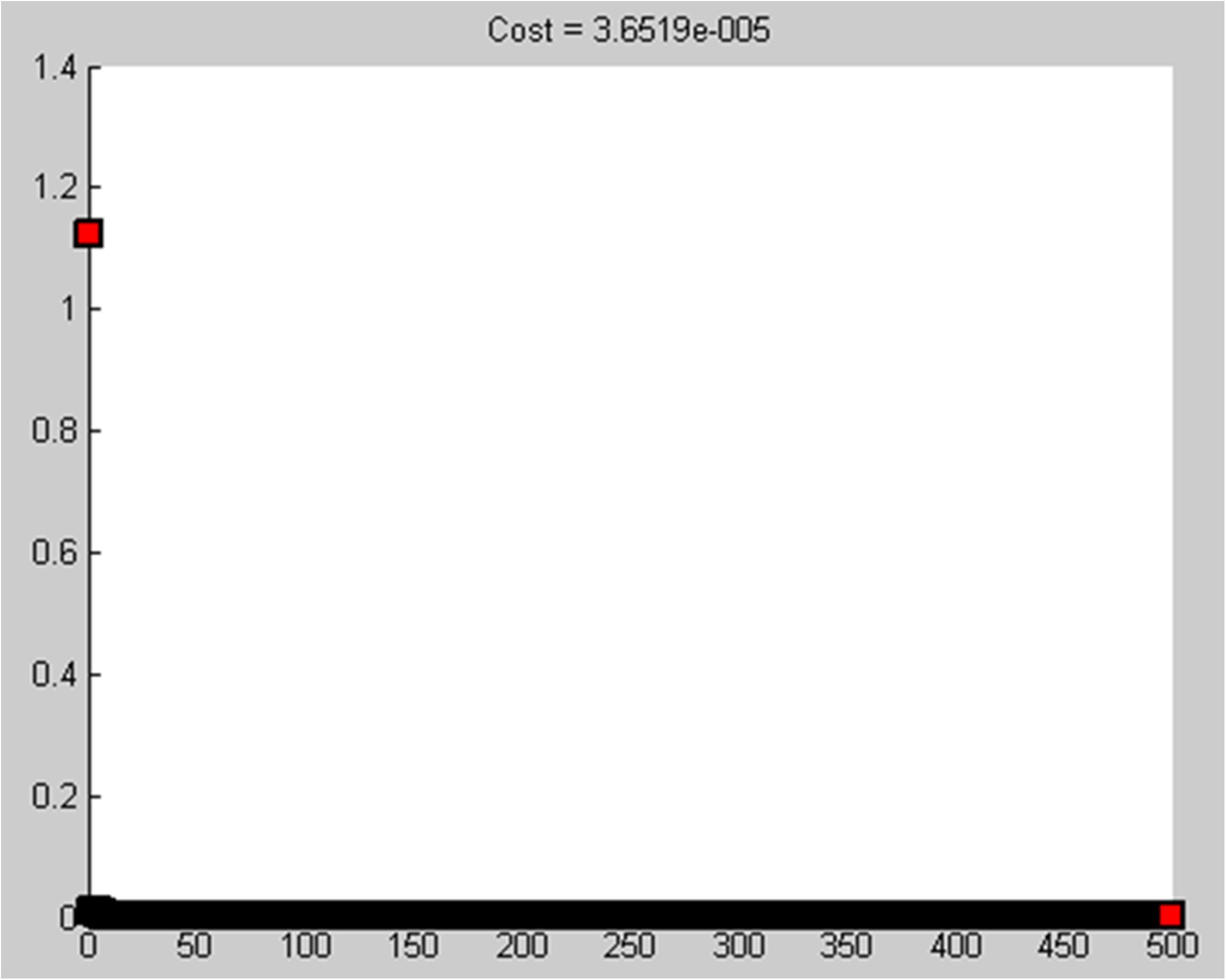

Supplement: Supplementary file 3 — Authors’ original file for figure 3 [file 40064_2013_395_MOESM3_ESM.tif]

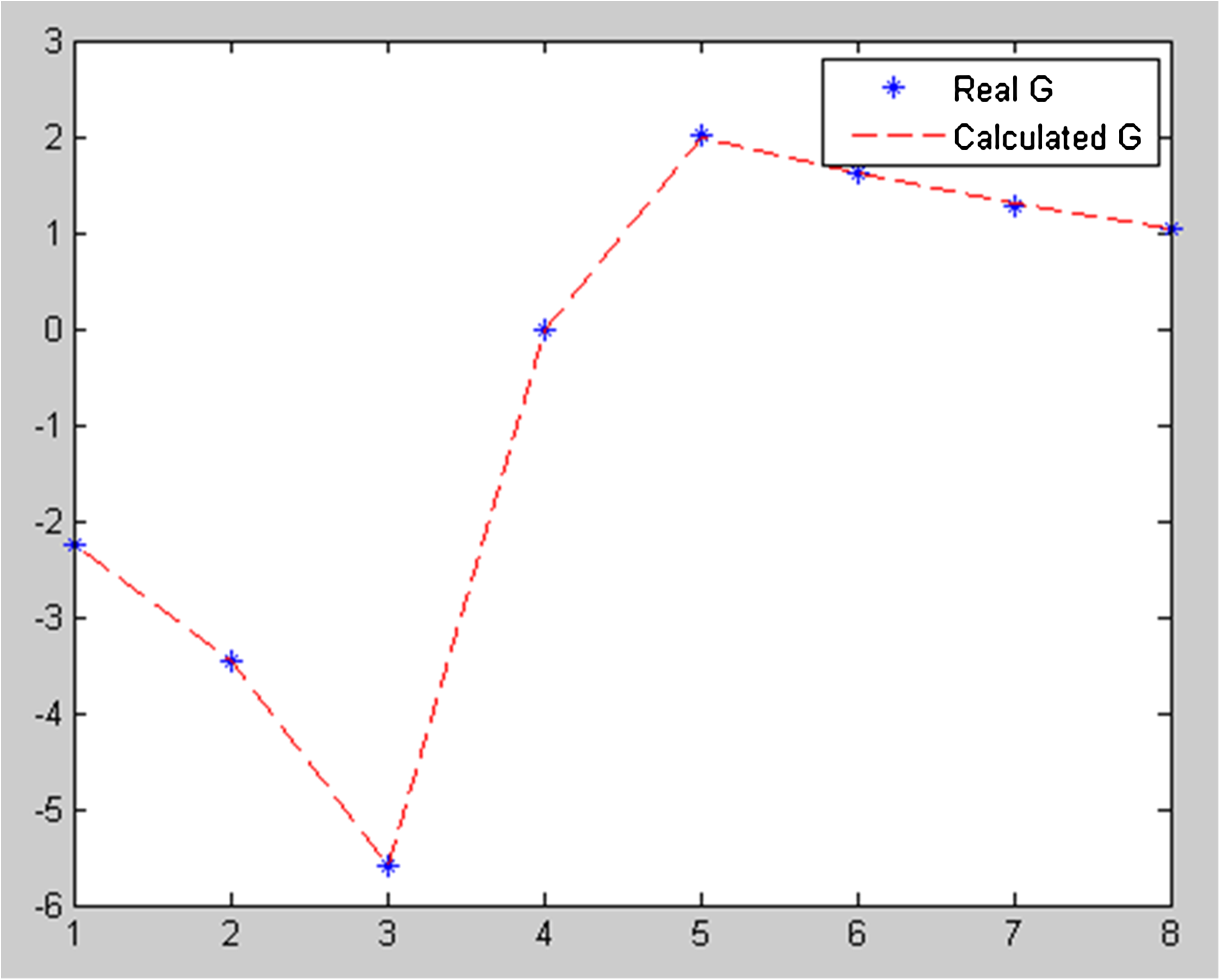

Supplement: Supplementary file 4 — Authors’ original file for figure 4 [file 40064_2013_395_MOESM4_ESM.tif]

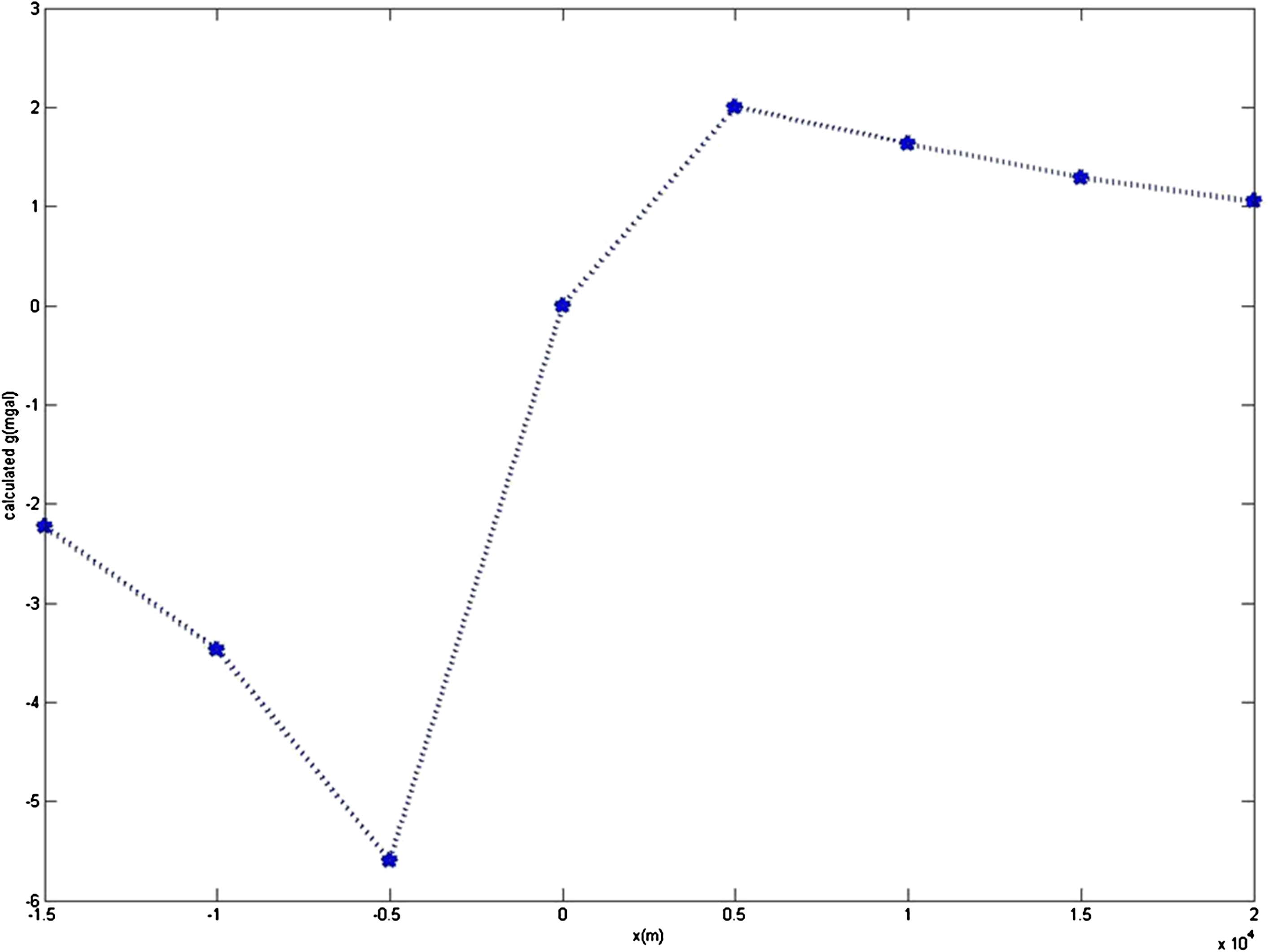

Supplement: Supplementary file 5 — Authors’ original file for figure 5 [file 40064_2013_395_MOESM5_ESM.tif]
